# Comparison of preemptive paracetamol, paracetamol-diclofenac & paracetamol-tramadol combination on postoperative pain after elective abdominal surgery under general anesthesia, Ethiopia: a randomized control trial study, 2018

**DOI:** 10.1186/s12871-020-01115-6

**Published:** 2020-08-04

**Authors:** Zemedu Aweke, Fetene Seyoum, Tewoderos Shitemaw, Derartu Neme Doba

**Affiliations:** 1 Department of Anesthesia, College of Medicine and Health Sciences, Dilla University, Dilla, Ethiopia; 2 Department of Anesthesia, Kotebe Metropolitan University, Menelik II Medical & Health Science College, Addis Ababa, Ethiopia

**Keywords:** Post-operative pain, Multimodal therapy, Preemptive analgesia, Pain management and laparotomy surgery

## Abstract

**Background:**

In the practice of postoperative pain management, pain is still poorly managed in low resource setting where the practice of epidural and opioid free analgesia is impractical. There has been a recent trend of combining different drugs and concept of preemptive analgesia but the therapeutic superiority remains understudied for postoperative pain management. The aim of this study is to assess postoperative analgesic effect of preemptive Paracetamol, Paracetamol-diclofenac and Paracetamol-tramadol combination in patients undergoing laparotomy surgery.

**Methods:**

Three-arm, randomized control trial study conducted on 63 patients undergone laparotomy surgery; group-P (paracetamol 1 g), group-PD (1 g + diclofenac 75 mg) and group-PT (paracetamol 1 g + tramadol 100 mg). The Numerical Rating Scale (NRS) pain rating system was used for this study. The primary endpoint of the study was total amount of analgesia consumption. Post-operative analgesic therapy [intravenous tramadol, 50 mg] were provided when patients complain of pain (request medication) or a numeric rating scale ≥4 was recorded. Secondary endpoint of the study were the time of first analgesic request and the intensity of the pain during 24 h post-op follow up period. Parametric data were analyzed using (ANOVA) and nonparametric data analyzed by Kuruska-Wallis H rank test. Chi-square test used for categorical variable. Statistical significance were sated at *p* value < 0.05 with a power of 80%.

**Results:**

The mean total tramadol consumption was significant higher in paracetamol group 250 ± 79.06 mg compared to paracetamol-diclofenac (173.81 ± 87.49 mg *p* = 0. 008) and paracetamol-tramadol (154.76 ± 70.54 mg *p* = 0. 001) group. Time to first analgesic request was significantly shorter within paracetamol group (87.62 ± 20.95 min) compared to paracetamol-diclofenac (103.01 ± 23.53 min *p* = 0.029) and paracetamol-tramadol (144.05 ± 14.72 min *p* < 0.001) group. There was statistically significant difference at 4th, 6th and 8th hour showing lower median pain score in paracetamol-tramadol group compared to paracetamol group.

**Conclusion:**

Preemptive combination of paracetamol-tramadol and paracetamol-diclofenac reduce total tramadol consumption and prolongs time to first analgesic request compared to paracetamol alone in patients undergoing laparotomy surgery.

**Trial registration:**

The study was retrospectively registered on 07 July 2019 at Pan African Clinical Trial Registry with the identification number of PACTR201908890749145. It was accepted on 14 August 2019.

## Background

Pain is defined as “unpleasant emotional and sensory experience due to actual or potential tissue damage”, according to international association for study of pain [1]. Studies demonstrates that a significant proportion of patients suffer moderate to severe intensity of pain in the immediate as well as early post-operative period after abdominal surgery [2, 3].

Poorly managed pain after surgery can negatively affect patients wellbeing on multiple levels such as hypertension, myocardial ischemia, arrhythmias, respiratory impairments, poor wound healings, chronic pain, deep vein thrombosis and risk factor for the development of chronic pain syndrome [4–6]. These negative outcomes lead to prolonged hospitalization resulting huge healthcare costs, patient’s financial expense as well as involvement of nursing care [7, 8].

Opioids are an alternative to postoperative pain management, although there are considerable side effects and addiction reported. However, in resource limited area, due to cost unaffordability, clinical setup and inaccessibility, managing pain through opioids is too difficult [9–11]. Epidural analgesia and opioid free analgesic techniques are also an alternative techniques but difficult to practice due to lack of resources in a low resources setting like Ethiopia.

Inadequate controlled postoperative pain remains a widespread problem despite the development of specialized acute pain management modalities, especially in developed country [12]. Indeed, current practice guidelines for perioperative pain management recommend the use of multimodal therapy [13, 14].

The adaptation of multimodal analgesic techniques as the standard method for prevention of pain in surgery is one alternative to improve the recovery process [14, 15]. Now a days administering a pain medication before surgery is one of the component in multimodal approach which is called preemptive analgesia. Preemptive analgesia is an antinociceptive treatment to block central nervous system hyperexcitability and leads to a reduced postoperative pain intensity and decrease the risk of postsurgical chronic pain [16, 17].

Despite this evidence-based approach to improve perioperative analgesia, the proportion of patients reporting moderate to severe pain after surgery has remained constant over the past decade [6, 18]. Consequently, for anesthetist in developing countries, providing effective postoperative pain management become practically difficult and open gate for investigating better intervention that are applicable in resource limited setups.Pre-emptive analgesia needs further investigation in order to find out optimal choice of drugs with minimum side effect, which are easy to administer and easily accessible and affordable [19]. Our study assess the combination of drugs which are opioid free analgesic (paracetamol & diclofenac) and the weakest opioid (Tramadol) that are easily available in countries with poor resources to use expensive analgesics. The aim of this study is by taking the pre-emptive analgesia model, to compare effectiveness of paracetamol, paracetamol-tramadol and paracetamol-diclofenac combination as a component of multimodal analgesia on postoperative pain. The study is done on patients undergoing elective abdominal surgery in a resource limited setup.

## Methods

A single blind randomized clinical trial was conducted at Hawassa Comprehensive Specialized Teaching Hospital from January 2018 to February 2019. Hawassa Comprehensive Specialized Teaching Hospital is one of the biggest government hospitals in Ethiopia functioning as a teaching and referral hospital since April 2005.

Ethical clearance approval was obtained from Dilla University institutional review board (IRB) with protocol number 007/18–08 on 4th September 2017. In addition, our study was retrospectively registered on July 07, 2019 at Pan African Clinical Trial Registry with the identification number of PACTR201908890749145. The purpose, importance and risk of the study was explained, written informed consent was obtained from each participant. Participants were informed that they can withdraw from the study without any restriction at any time. This study is adhered to CONSORT guidelines.

Patients undergone elective laparotomy surgery at Hawassa Comprehensive Specialized Teaching Hospital in the course of data collection period were included in the study. Patients with age greater than 18 years old and ASA physical status class I–II were included in the study. The exclusion criteria’s were concomitant medical or psychiatric problems which prevent completion of the follow-up, acute or chronic pain diagnosis, history of upper gastrointestinal bleeding related to previous NSAID therapy, comorbidities (Anemia, DM, HTN, Arthritis), moderate or severe renal impairment (serum creatinine > 1.6 mg/dl), known asthmatic patient, history of alcohol, opiate or other drug abuse, use of NSAIDs or Paracetamol within 24 h of surgery and participants with known allergies for the study drugs.

### Sample size and sampling procedure

Before this study begun, we conducted a pilot study for estimating a sample size. The Sample size estimation were calculated using a priori power analysis (G Power version 3.1.9.2) based on the pilot study results. The outcomes measure for this study were the mean total analgesic consumption between groups over 24 h, time to first analgesic request and numeric rating scale (NRS) score between groups.

Based on largest sample size, the mean total analgesic (tramadol) consumption was used to estimate the sample size. The observed mean total tramadol consumption from the pilot study was μ1 = 215 ± 80 mg μ2 = 170 ± 60 mg μ3 = 155 ± 52 mg, SDpooled = 51.33. A priori power analysis for a one-way ANOVA with 3 groups was conducted to determine sample size using an alpha = 0.017(Controlling for the probability of a Type I error using a Bonferroni correction). To allow for comparisons of the three groups, a sample of 19 subject per group would have 80% power to detect the difference of the mean total tramadol consumption between groups. The calculated sample size was 57; by adding 10% attrition rate and assuming balanced design the total sample size become 63. The results of that pilot study were not included in the present analysis, and none of the patients from the pilot study were included in the present study.

**Fig. 1:**
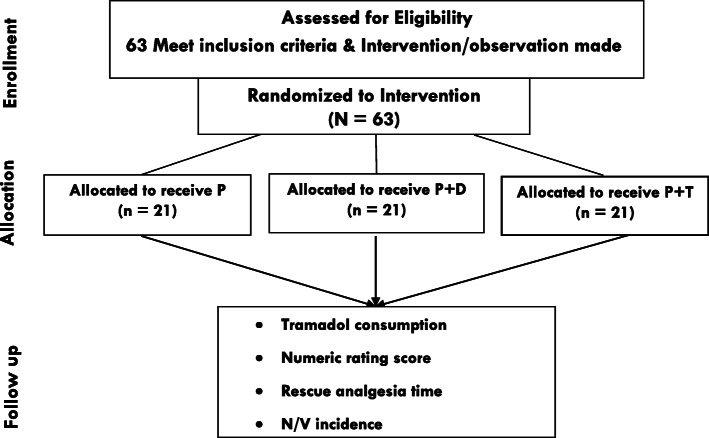
Enrollment chart for patients scheduled for Laparotomy

By reviewing a five-month report, a systemic random sampling method was applied with the probability of 50% to be included in the study (k = ½). Considering the sequential patients scheduled for laparotomy surgery, a random start was used to select every eligible participants. After then, the patients were allocated to one of the three groups randomly with sealed non-transparent envelope containing the name of the group. (Fig. 1).

### Data collection procedures

A day prior to the day of surgery, a staff member not involved in data collection performed preoperative assessment and took informed consent. All patients who were scheduled for elective laparotomy, fulfilled the inclusion criteria and volunteer to take part in the study were instructed by data collectors on how to self-report pain using the eleven point Numerical Rating Scale (NRS), a score from 0 to 10. The instruction on how to self-report pain was repeated for study participant on the morning of the surgery.

At midnight, the day before surgery, oral paracetamol (1 g) with sips of water was given for all three groups. At the morning of surgery (60 Minutes before surgery) 1 g of oral (PO) paracetamol (Group P) or 75 mg of Intra muscular (IM) diclofenac (Group- PD) or 100 mg of Intravenous (IV) tramadol (Group- PT) was provided in accordance with the random allocation of the patient to specific group. The time elapsed between the mid night oral paracetamol provided and the morning dose of preemptive analgesia was 8–10 h. The prepared drug was given by the personnel anesthetist assigned to each case who were not involved in the study. Owing to the nature of the study and the study site, blinding was not possible for the patient and anesthetist delivering the interventions.

When the study participant arrived at the waiting room, all participants were premedicated with dexamethasone (8 mg), diazepam (5 mg) and cimetidine (400 mg). Preoperatively, baseline vital sign, the presence and severity of pain (NRS for pain) and demographic data were obtained.

**Fig. 2:**
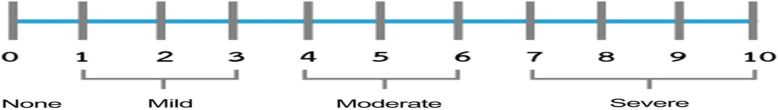
Numerical pain rating scale (NRS), adopted from the National Initiative on Pain Control™ (NIPC™)

The Numerical Rating Scale (NRS) pain rating system was used for this study. The NRS showed higher compliance rates (across cultures and languages), better responsiveness, easier to use and good applicability than Visual Analogue scale (VAS) in different studies [20–22]. In addition the NRS was the instrument of choice in an age-mixed population making it preferable for this study [21]. (Fig. 2).

The same general anesthesia protocol was applied in all groups without any regional or neuraxial anesthesia. Standard ASA monitors applied to all the patients that include a pulse-oximetry, electrocardiography, noninvasive blood pressure, and a temperature monitor. Induction of general anesthesia were achieved with Propofol at 2.5 mg/kg- ketamine 0.3 mg/kg (analgesia dose), fentanyl (50 μg) and tracheal intubation facilitated by succinylcholine (2 mg/kg) after 3 min’ of pre-oxygenation.

Anesthesia was maintained by using isoflurane with oxygen (100%) mixture and a low gas flow (3 L/minute) in accordance to clinical needs/assessment of the depth of anesthesia. The patient’s mechanical ventilation parameters were adjusted based on the patient’s age and weight. Neuromuscular blockage was maintained with Vecuronium. At the end of the surgery, residual neuromuscular block were antagonized with atropine 0.2 mg/kg and neostigmine 0.05 mg/kg. After surgery, participants were transferred to the PACU with adequate emergence from anesthesia.

Outcome data was collected by anesthetists who were blinded to the treatment allocation. Observations begun at post anesthesia care unit. All patients were assessed using systematically structured questionnaire by four trained data collector (BSc Anesthetist with minimum of 3 years’ experience). Furthermore, MSc anesthetist was assigned to assist and supervise data collectors. All patient were asked to rate pain intensity on standard numeric rating scale range from 0(no pain) to 10(worst possible pain) at rest (static NRS) and voluntary cough (dynamic NRS).

The pain intensity was rated as mild (NRS: 0–3), moderate (NRS: 4–6), and severe (NRS: 7–10). The NRS score were recorded at recovery after the patient is fully awake from anesthesia, at the 2nd, 4th, 6th, 8th, 12th and 24th hour. Twenty-four-hour postoperative analgesia consumption, time to first analgesia request and incidence of nausea vomiting were recorded. The supervisor checked each questionnaire daily with further cross check by principal investigator for completeness and consistency of data.

In case of nausea or vomiting, intravenous metoclopramide 10 mg and dexamethasone 4 mg was administered. For the first 24 h post-operative analgesic therapy were provided when patients complain of pain (request medication) or a numeric rating scale ≥4 was recorded. The analgesic drug administered were intravenous tramadol (50 mg) according to the hospital protocol. After 24 h post-operative analgesic therapy continue according to the hospital protocol. The post-operative analgesia drug provided to patients were only tramadol since morphine was not available at the study site during the study time.

### Operational definition

- **Preemptive analgesia** is anti-nociceptive treatment that starts before surgery and prevents the establishment of peripheral and central sensitization [23].
- **Postoperative pain**: the presence of pain in the postoperative period was defined as a pain occurring in a surgical patients following a procedure [24].
- **Numerical pain rating scale (NRS):** is a valid is a method of pain assessment where patients are asked to score their pain ratings on a scale of 0–10, corresponding to current, best, and worst pain experienced over the 24 h. The median value will be used to represent patient’s level of pain [25]. (Fig. 2)
- **Total analgesia consumption**: is total amount of analgesic drugs in milligrams used in the first 24 h after the operation.
- **Laparotomy/Abdominal surgery**: any operation that involve opening the abdominal cavity for Diseases affecting the abdominal cavity through the sheath of the rectus abdominis muscles/ midline incision surgery.
- **Time to first analgesic request**: is a time in minutes measured from the end of procedure to time when patient request analgesics.

### Data analysis and interpretation

The statistical analyses were performed using SPSS V 23 software. The data was tested for normality using Shapiro–Wilk normality test and homogeneity of variance by Levene’s test. Analysis of variance (ANOVA) and Kruskal–Wallis H test were used for normally distributed continuous data and non-normally distributed or non-parametric data respectively. If the ANOVAs or Kruskal–Wallis H test were significant, then Tukey post hoc test was used to compare one group with the others. Categorical data were analyzed using the Pearson Chi-squared test. Data was expressed as a mean and standard deviation (SD) or Median (Q1-Q3). A *P-value* < 0.05 with power of 80% was considered as a statistically significant.

## Results

### Socio-demographic data

Sixty-three patients participated in this study. There was no significant difference among the three groups with regard to age, gender, weight, operation duration, anesthesia duration, ASA physical status and baseline preoperative pain (*p* value > 0.05) as depicted in Table 1.

**Table 1:** socio demographic characteristics of patients who undergo elective laparotomy surgery at Hawassa compressive specialized hospital

	Group P	Group PD	Group PT	*P*-value
Age (year)^a^	41.67 ± 7.52	45.33 ± 7.72	42.10 ± 9.07	0.286
Gender (M /F)^b^	10/11	12/9	8/13	0.466
Weight (kg)^a^	58.28 ± 3.47	57.52 ± 3.54	59.26 ± 3.67	0.300
ASA I / II^b^	16/5	18/3	17/4	0.734
Operation duration (min)^a^	118.10 ± 23.05	112.38 ± 19.34	114.05 ± 17.51	0.640
Anesthesia duration (min)^a^	134.76 ± 22.28	129.76 ± 19.97	132.14 ± 17.72	0.723
Type of surgery^b^				
Gastrointestinal(n)	13(61.9%)	12 (57.2%)	11 (52.3%)	0.436
Gynecological(n)	8 (38.1%)	9 (42.8%)	10 (47.7%)	
Surgical incision length	15 (14–16)	15 (15–17)	14 (15–17)	0.765

Data are given as mean ± SD or Median (Q1-Q3) or Case number/ proportion (n/%), (ANOVA, Kruskal-Wallis test and Tukey test, Chi-Square Test)

^a^Mean ± SD

^b^Case number (proportion)

### Postoperative numeric rating scale score at rest and during movement

**Fig. 3:**
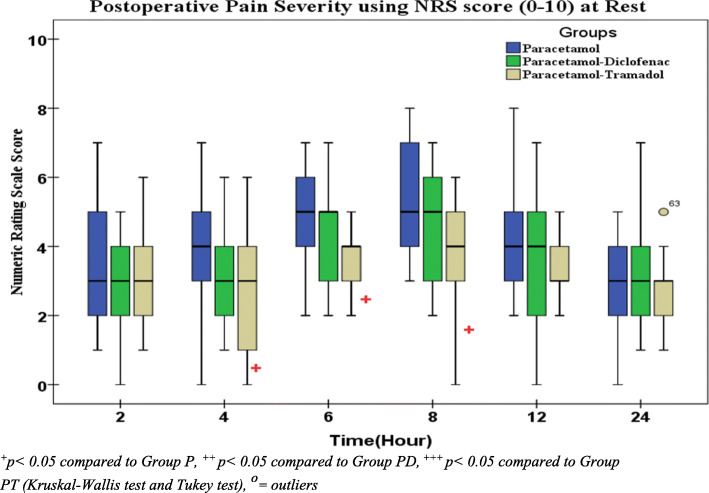
Comparison of postoperative pain using 11 point NRS score (0–10) at rest

The Kruskal-Wallis test showed that median NRS score were not significant at 2nd, 12th and 24th hours between the three groups (*p* > 0.05). There was statistically significant difference on the NRS score at the 4th, 6th and 8th hour between groups (*p = 0*.032, 0.022 and 0.007 respectively). Post hoc analysis shows a significantly reduced NRS score in the paracetamol-tramadol combination (PT group) than the paracetamol group (P group) at rest with *p* = 0.25, 0.013 and 0.002 respectively. There was no statistically significant difference between the two combination group (PD group & PT group) at all time during 24 h (*p* > 0.05). However, NRS scores of paracetamol-tramadol group were lower than paracetamol-diclofenac at all time. Again there was no statistically significant difference results between paracetamol-diclofenac combination and paracetamol group at all time during 24 h (*p* > 0.05). But, NRS scores of paracetamol-diclofenac group were lower than paracetamol group at all time (Fig. 3*).*

**Fig. 4:**
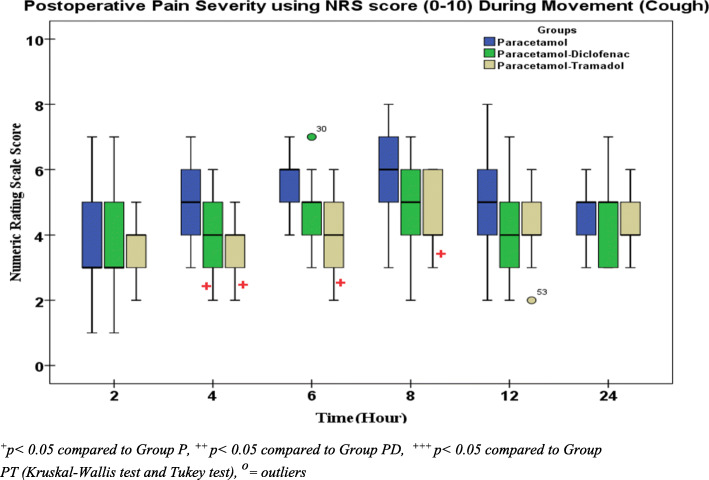
Comparison of postoperative pain using 11 point NRS score (0–10) on Movement

The Kruskal-Wallis test showed that median NRS score were not significant at 2nd, 12th and 24th hours (*p* > 0.05) between three groups during coughing. There was statistically significant difference results at the 4th, 6th and 8th hour between groups (*p =* 0.005, 0.008 and 0.007 respectively). Post hoc analysis shows significant reduction in NRS score during coughing in the paracetamol-tramadol combination group when compared to paracetamol alone group with *p* value (*p =* 0.003, 0.003 and 0.011 at 4th, 6th and 8th hour respectively). A significant reduction of NRS during coughing was also recorded in the paracetamol-diclofenac combination group compared to paracetamol alone at 4th hour (*p = 0.015*). There was no statistically significant difference results between the two combination group (PD group & PT group) at all time during 24 h (*p* > 0.05). However, NRS scores of paracetamol-tramadol group were lower than paracetamol-diclofenac at all time (Fig. 4*).*

### Time to first analgesia request

The time to first analgesia requirement in the paracetamol group (M ± SD: 87.62 ± 20.95 min) was significantly shorter than in the paracetamol-diclofenac group (103.01 ± 23.53 min, *p* = 0.029) and paracetamol-tramadol groups (144.05 ± 14.72 min, *p* < 0.001). Likewise first analgesia requirement in the paracetamol-diclofenac was significantly shorter than paracetamol-tramadol group (*p* < 0.001) (Table 2).

**Table 2:** Time to first analgesic request in minutes and total analgesia consumption over 24 hours

	Group P	Group PD	Group PT	*P*-value
**First analgesic requirement** **time (minutes)**^a^	87.62 ± 20.95	103.01 ± 23.53^+^	144.05 ± 14.72^+, ++^	< 0.001
**Total analgesics consumption**				
o Tramadol in mg (IV)^a^	250 ± 79.06	173.81 ± 87.49^+^	154.76 ± 70.54^+^	0.001

Data are given as mean ± SD (ANOVA and Tukey test)

^a^Mean ± SD

^+^*p* < 0.05 compared to Group P, ^++^*p* < 0.05 compared to Group PD

### Total cumulative postoperative analgesia consumption within 24 h

There was statistically significant difference in mean total tramadol consumption within 24 h postoperatively between the groups as shown in Table 2. Post hoc analysis of total tramadol consumed in 24 h showed significantly higher in paracetamol group compared to paracetamol-diclofenac group (*p* = 0.008) and paracetamol-tramadol group (*p* = 0.001). However, no significant difference between paracetamol-diclofenac and paracetamol-tramadol groups (Table 2).

### Incidence of nausea and vomiting

The incidence of nausea and vomiting over 24 h was 23.8%. The proportions of patients with nausea and vomiting was lower (19%) in PD group compared to P and PT group (X^2^ = 0.525) but not significant (*P* = 0.769). No serious complications or life-threatening events occurred in either group.

## Discussion

Our study showed that the total tramadol consumption was lower in both paracetamol-diclofenac and paracetamol-tramadol, combination over 24-h compared to paracetamol alone group. The mean total tramadol consumption was 154.76 ± 70.54 mg in paracetamol-tramadol group compared to 173.81 ± 87.49 mg in paracetamol-diclofenac group and 250 ± 79.06 mg in paracetamol group (*p =* 0.001).

In a study by Samimi et al. the mean total morphine consumption in patients receiving the combination of paracetamol-diclofenac over the first 24 h was significantly lower (13.9 ± 2.7 mg) compared to diclofenac group (20.1 ± 3.6 mg) with *p <* 0.05. Our study used the weakest opioid (tramadol) for post-operative pain management. To compare our result with the above study by Samimi et al. using opioid conversion factor of tramadol to morphine (0.1), paracetamol-diclofenac group in our study consumed an estimated mean of 17.4 mg morphine [26].

Study done by Montgomery et al, showed total postoperative morphine consumption in paracetamol-diclofenac group (18.5–35.8) (mean, 95% CI) was lower than paracetamol group (36.1–53.6) with *p < 0.01*. Montgomery and colleagues reported that the use of this combination has been shown to reduce the amount of morphine required by about one-third compared with paracetamol alone in women undergoing elective abdominal gynecological procedures [27]. These result was also in line with the study done by Moussa and Riad that assessed total opioid consumption between paracetamol-diclofenac combination and paracetamol alone that found significantly lower total morphine consumption in paracetamol-diclofenac combination: mean (SD) 2.9 (1.5) mg compared to paracetamol alone: 5.5 (1.5) mg with *p <* 0.01 [28].

This study demonstrated a non-significant difference in median (IQR) NRS at 2nd, 12th, and 24th hour between groups. Median (IQR) NRS score significantly lower in paracetamol-tramadol group at 4th, 6th and 8th hours compared to the paracetamol group (*p* = 0.032, *p* = 0.022 and *p* = 0.007 respectively). This result was comparable with the result of a study done by Solmaz and Kovalak which found that significantly lower VAS in acetaminophen- tramadol combination compared to acetaminophen alone at 1st and 2nd hour (2.10 ± 1.48 Vs. 4.75 ± 3.05 *p* = 0.030 and 3.30 ± 1.71 Vs. 6.10 ± 1.86 0.020, respectively). The difference in VAS between the groups disappeared in the second hour (3.45 ± 1.63 Vs. 3.95 ± 1.43 *p* = 0.129) [29].

In contrary to our finding, pain score (VAS) reported by Samimi et al. showed statistically significant difference among paracetamol-diclofenac combination, diclofenac group and placebo groups during the first 24 h (*p* < 0.05). The possible explanation for this difference in result might be due to pain management practice in study set up or the use of additional analgesic agent in perioperative period [26].

According to our study the mean time to first analgesia request in minutes were shorter in the paracetamol group (M ± SD: 87.62 ± 20.95 min) compared to PD group (103.01 ± 23.53 min, *p* = 0.001) and PT groups (144.05 ± 14.72 min, *p* < 0.001). This finding is in line with study done by Samimi et al. (*p* < 0.05) [26].

Combining several analgesic modalities into a single analgesic regimen often referred to as multimodal analgesia, may hold the greatest promise for limiting sensitization of the nervous systems by noxious stimuli [30]. Effective pre-emptive analgesic techniques provide multi-level interruption of nociceptive inputs, increased pain threshold and decreased activation of nociceptors [23, 31]. Our results showed that combination therapy was superior to paracetamol alone. The low values of the pain scores in combination groups may be explained by decreased excitability in the central nervous system through blockade of nociceptive stimuli at different site before tissue damage.

The current study has certain limitations, including lack of control over the confounding factor like incision size and participation of different anesthetist and surgeon. In addition, one of the potential study design drawbacks is the shorter duration of postoperative follow up. The discussion part is limited by the unavailability of adequate studies to compare with our result.

## Conclusion

Analgesic drug combination of paracetamol-tramadol and paracetamol-diclofenac reduce total tramadol consumption and prolongs time to first analgesic request compared to paracetamol before surgical intervention alone in patients undergoing laparotomy surgery. The paracetamol-tramadol combination seems superior to paracetamol-diclofenac combination.

## Data Availability

The data used in this study was collected by trained data collectors and authors are willing to share the data upon request from peer researchers. To request the data, Contact the first author (Zemedu Aweke, zemeduAwoke@yahoo.com).

## Abbreviations

ASA: American Society of Anesthesiologists
CI: Confidence Interval
GA: General Anesthesia
NRS: Numerical Rating Scale
PACU: Post Anesthesia Care Unit
SPSS: Statistical Package for Social Sciences
VAS: Visual Analogue scale
